# Pattern of neurological recovery in persons with an acute cervical spinal cord injury over the first 14 days post injury

**DOI:** 10.3389/fneur.2023.1278826

**Published:** 2023-12-19

**Authors:** Nader Fallah, Vanessa K. Noonan, Zeina Waheed, Raphaele Charest-Morin, Charlotte Dandurand, Christiana Cheng, Tamir Ailon, Nicolas Dea, Scott Paquette, John T. Street, Charles Fisher, Marcel F. Dvorak, Brian K. Kwon

**Affiliations:** ^1^Praxis Spinal Cord Institute, Vancouver, BC, Canada; ^2^Department of Medicine, University of British Columbia, Vancouver, BC, Canada; ^3^Department of Orthopaedics, Vancouver Spine Surgery Institute, University of British Columbia, Vancouver, BC, Canada; ^4^International Collaboration on Repair Discoveries (ICORD), University of British Columbia, Vancouver, BC, Canada

**Keywords:** acute cervical spinal cord injury, motor recovery trajectory, baseline neurological assessment, upper-extremity motor score, total motor score

## Abstract

**Introduction:**

Following a traumatic spinal cord injury (SCI) it is critical to document the level and severity of injury. Neurological recovery occurs dynamically after injury and a baseline neurological exam offers a snapshot of the patient's impairment at that time. Understanding when this exam occurs in the recovery process is crucial for discussing prognosis and acute clinical trial enrollment. The objectives of this study were to: (1) describe the trajectory of motor recovery in persons with acute cervical SCI in the first 14 days post-injury; and (2) evaluate if the timing of the baseline neurological assessment in the first 14 days impacts the amount of motor recovery observed.

**Methods:**

Data were obtained from the Rick Hansen Spinal Cord Injury Registry (RHSCIR) site in Vancouver and additional neurological data was extracted from medical charts. Participants with a cervical injury (C1–T1) who had a minimum of three exams (including a baseline and discharge exam) were included. Data on the upper-extremity motor score (UEMS), total motor score (TMS) and American Spinal Injury Association (ASIA) Impairment Scale (AIS) were included. A linear mixed-effect model with additional variables (AIS, level of injury, UEMS, time, time^2^, and TMS) was used to explore the pattern and amount of motor recovery over time.

**Results:**

Trajectories of motor recovery in the first 14 days post-injury showed significant improvements in both TMS and UEMS for participants with AIS B, C, and D injuries, but was not different for high (C1–4) vs. low (C5–T1) cervical injuries or AIS A injuries. The timing of the baseline neurological examination significantly impacted the amount of motor recovery in participants with AIS B, C, and D injuries.

**Discussion:**

Timing of baseline neurological exams was significantly associated with the amount of motor recovery in cervical AIS B, C, and D injuries. Studies examining changes in neurological recovery should consider stratifying by severity and timing of the baseline exam to reduce bias amongst study cohorts. Future studies should validate these estimates for cervical AIS B, C, and D injuries to see if they can serve as an “adjustment factor” to control for differences in the timing of the baseline neurological exam.

## Introduction

The clinical evaluation of acute traumatic spinal cord injury (SCI) utilizes the widely accepted International Standards for the Neurological Classification of SCI (ISNCSCI) examination to characterize the degree of neurological impairment (1). This exam provides a standardized way to report the level and severity of injury and has been used to predict neurological recovery and outcome (2). It is recommended that the ISNCSCI exam is done following the SCI (1, 3). However, the challenge in the acute setting is that the SCI itself is evolving from the moment that it happens. Many patients, for example, will describe a period of complete paralysis at the scene of the accident when the initial injury occurs, with subsequent improvement to varying degrees of incomplete motor/sensory recovery observed in the ensuing hours and days. Because this is a dynamic process, how one interprets recovery will invariably be influenced by when the neurological assessment is actually done (i.e., when the “snapshot” of neurological impairment is actually taken). For example, if a patient begins at the scene of the accident (prior to any formal ISNCSCI examination) with a motor score of 0, and at 1-month post-injury has a motor score of 25, how one interprets this amount of recovery will depend upon when the first formal ISNCSCI examination actually occurred. Perhaps this patient had a motor score of 5 on arrival in hospital 4 h later, and then by the time magnetic resonance imaging (MRI) was conducted and the patient was taken to the operating room, ~12 h post-injury, the motor score was 10. If the clinical team was able to assess an ISNCSCI examination at 4 h post-injury, it would be interpreted that at 1 month the recovery was 20 points. But if the ISNCSCI was performed at 12 h, motor recovery would be deemed to be 15 points (25% less), just due to the timing of the baseline examination.

Studies investigating neurological recovery following SCI vary in terms of when the baseline neurological exam is conducted and have ranged from 2 h up to 30 days (4–14) (see Table 1). Practically, the precise timing of the examination is often not documented and this lack of recorded time further complicates the understanding of neurological recovery following the initial assessment. Because the time of the baseline neurological assessment is not standardized in registries, this issue may confound studies where investigators use specific inclusion criteria for the intervention group but use a control group from a registry where the timing of the first neurological exam varies from one day to one-month post injury. Bias can be introduced into the analysis if exams performed earlier post-injury (i.e., before the possibility of spontaneous neurological recovery) are grouped with later examinations that may have been taken after or during spontaneous neurological recovery (15). In this case, participants in the “earlier exam” group may falsely exhibit greater neurological improvement in response to the intervention than the “later exam” group. It is also important that the examiners are trained and have experience to ensure the exam results are reliable and valid (16, 17).

**Table 1:** Comparing distribution of first neurological exam in cervical SCI studies.

**References**	**Number of participants**	**First ISNCSCI exam post injury (approximate time)**	**Follow-up ISNCSCI exam**	**Neurological level of injury (approximate time)**	**Neurological severity (AIS)**
Maynard et al. (4)	114	72 hours	1 year	Frankel classification	A–D: based on Frankel classification
Marino et al. (5)	482	7 days	1 year	C1–L5	A–D: based on Frankel classification and AIS
Burns et al. (6)	103	48 hours	1 year	Not available	A–D
Fawcett et al. (7)	Review paper so NA	30 days	1 year	C1-L5	A–D
Curt et al. (8)	1140	14 days	48 weeks	Tetraplegic, paraplegic	A–D
Van Middendorp et al. (9)	161	15 days	6 months−1 year	C1–T11	A–D
Marino et al. (10)	125	7 days	1 year	C1–C8	A–D
Steeves et al. (11)	305	72 hours−7 days	1 year	C4–C7	A
Kirshblum et al. (12)	187	30 days	1 year	C1–L5	A
Evaniew et al. (13)	85	48 hours	1 year	C1–T1	A
Balbinot et al. (14)	748 440 (subset)	4 weeks 7 days	48 weeks for all	C1–C8	A–D

AIS, American Spinal Injury Association (ASIA) Impairment Scale; ISNCSCI, International Standards for the Neurological Classification of Spinal Cord Injury; NA, not applicable.

Furthermore, most of the evidence on neurological recovery is based on a cross-sectional or longitudinal study design with only a few time points (e.g., at admission and scheduled follow-ups). Given the nature of neurological recovery, a longitudinal study design that includes multiple data points (e.g., on admission, following surgery, on admission to rehab, at discharge) temporally recorded would help describe neurological recovery following injury and allow researchers to more appropriately adjust for the differences between groups (e.g., cases and controls). Finally, earlier work by our research group and others has highlighted the importance of controlling for heterogeneity of SCI by appropriately stratifying study participants into categories by both neurological severity and level of injury, recording the number of study participants, and reporting the mean baseline motor scores for each study participant category (15, 18).

To understand the trajectory of neurological recovery following SCI, we examined the relationship between the timing of neurological assessment and motor recovery over the first 14 days post injury using a longitudinal study design in persons with cervical SCI, as this is the average time frame reported in SCI studies (4–6, 8–11, 13, 19). The specific study objectives were to: (1) describe the pattern and amount of motor recovery in persons with an acute cervical SCI over the first 14 days (including taking into consideration the neurological level and severity of the SCI); and (2) evaluate if the timing of the first neurological examination over the first 14 days biases the amount of motor recovery observed.

## Materials and methods

### Study design

A retrospective cohort analysis using a longitudinal study design was used. For this study, we focused on motor recovery following cervical SCI, given this is often an outcome used for SCI clinical trials (20, 21).

### Study cohort

Patients were enrolled in the Vancouver site of the Rick Hansen SCI Registry (RHSCIR), a pan-Canadian prospective observational registry of 30 major acute and rehabilitation hospitals, between 2004–2012. Full details of the RHSCIR have been described elsewhere (22). Eligibility for the study included participants with an acute cervical SCI (C1–T1) who had a minimum of two neurological exams with upper-extremity motor score (UEMS), total motor score (TMS) and American Spinal Injury Association (ASIA) Impairment Scale (AIS) data conducted within the first 2 weeks of injury (at least one in each week except AIS which was just for the first exam), and a final exam with the same data elements (UEMS, TMS, AIS) within RHSCIR.

### Participant, injury and care management data variables

Demographic and injury data on RHSCIR participants included age, sex, mechanism of injury (i.e., assault, fall, sport, transport, or other) (23), a total count of medical comorbidities based on the Charlson Comorbidity Index (24, 25), and additional injuries to other body regions using the Injury Severity Score (ISS) (26). Data describing the provision of care consisted of the time to the acute hospital (Vancouver General Hospital), spine procedures (number receiving surgery, timing of surgery), admission to rehabilitation (GF Strong Rehabilitation Center), and acute as well as rehabilitation length of stay.

Neurological impairment was assessed using the ISNCSCI (1, 3, 27). The date and time of these examinations (recorded in days post injury) were obtained from RHSCIR. The neurological exams were conducted by the clinical team who are trained on how to complete the ISNCSCI exam including physical therapists, nurses, spine residents/fellows and spine surgeons. Data from the ISNCSCI included the neurological level of injury (NLI), AIS to describe the injury severity, and the UEMS and TMS. The AIS classifies persons with SCI as having a motor-sensory complete injury (AIS A), a motor complete and sensory incomplete injury (AIS B), or a motor-sensory incomplete injury (AIS C or AIS D). The UEMS includes five muscles groups scored out of 5 per extremity, for a total score of 50 and a TMS of 100 (1, 3). The AIS grade assignment was verified using the Praxis ISNCSCI Algorithm which provides an AIS grade based on the motor and sensory data, including voluntary anal contraction (VAC) and deep anal pressure (DAP) (2). Neurological severity (AIS A, B, C, D), level of injury (high cervical C1–C4; low cervical C5–T1), the UEMS and TMS were obtained. A hospital chart review was conducted to obtain additional neurological exam data during the individuals' acute in-patient admission.

### Statistical analysis

First, a descriptive analysis of the data was conducted. Continuous variables were reported using mean and standard deviation, and categorical variables were described using a frequency (percentage). Missing data was not imputed. Trajectories of neurological recovery using motor scores (UEMS, TMS) from the first day up to 14 days post injury and a final exam prior to discharge, were created for subgroups AIS A, B, C and D.

Data was then stratified by neurological severity (AIS A, B, C, D) and level of injury (high cervical C1–C4; low cervical C5–T1) to determine if there were any differences in recovery between these groups. To explore the pattern and amount of motor recovery over time (up until the last exam) as well as the effect of when the exam was conducted over 14 days post injury (at least one exam was done in the first week and at least one exam was done in the second week), a linear mixed-effect model was used. In all of the linear mixed-effect models, a fixed-effects model was used first and the complexity of the model was increased in steps by adding random effects and additional variables [i.e., time (linear form), time^2^ (quadratic form), AIS, level of injury, and motor score (UEMS, TMS)]. This process was continued until there was no improvement in the log-likelihood value, AIC and BIC goodness of fit criteria. The rate of conversion of AIS grade from the first to final assessment was also calculated and compared to the literature. A *p*-value of < 0.05 was considered statistically significant. All statistical analyses were performed using SPSS (version 27) and Rx64 (version 3.3).

## Results

**Figure 1:**
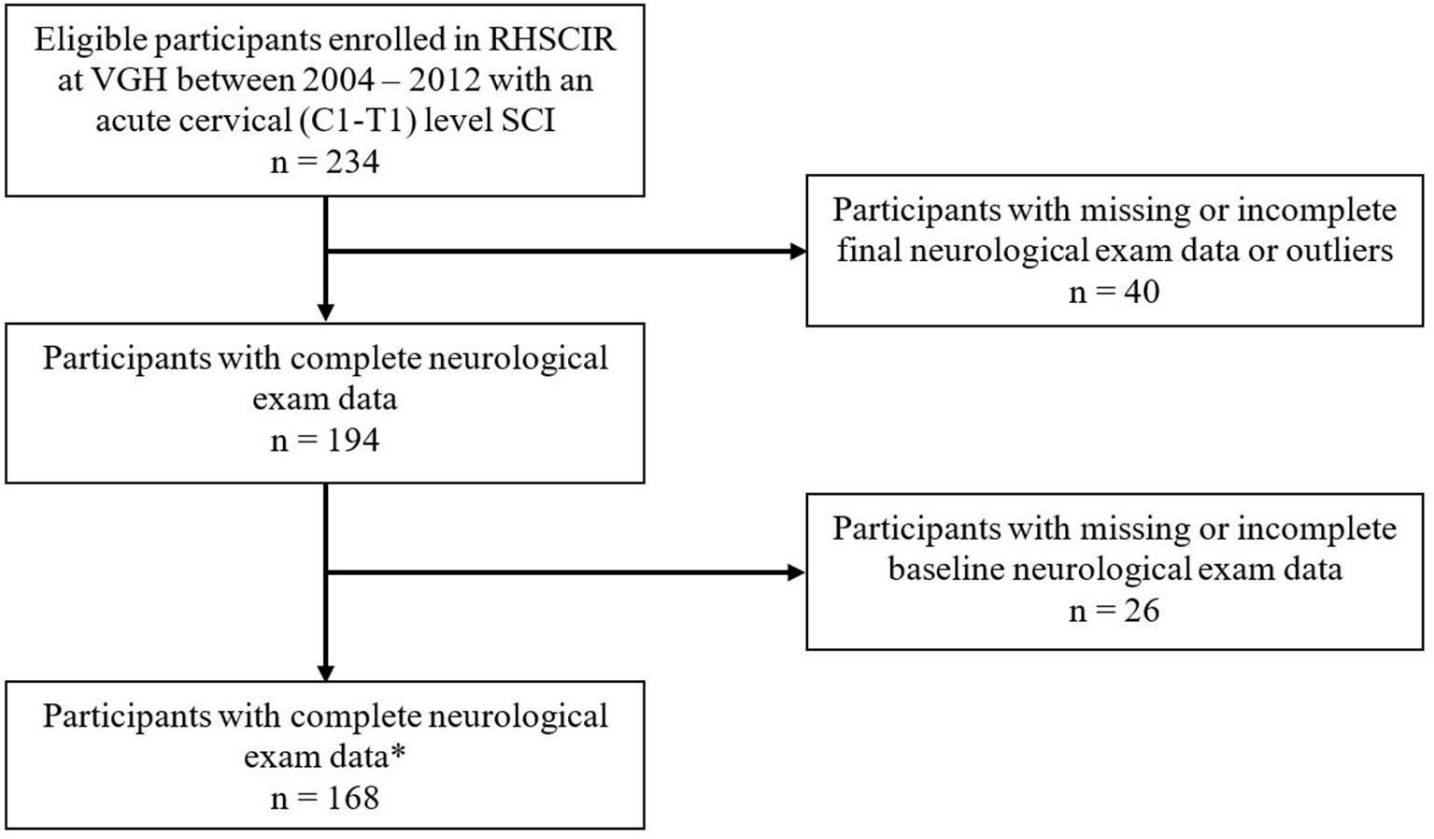
Study Consort Diagram of 849 individuals who were assessed for eligibility. *Neurological exam data included: UEMS, upper-extremity motor score; TMS, total motor score; and AIS, American Spinal Injury Association (ASIA) Impairment Scale. VGH, Vancouver General Hospital.

Between 2004 and 2012, a total of 849 individuals were admitted to Vancouver General Hospital, and enrolled in the Rick Hansen Spinal Cord Injury Registry (RHSCIR), among these participants, 234 individuals had cervical spinal cord injury spanning from C1–T1. Sixty-six participants were excluded because they did not have at least two neurological examinations, resulting in a study cohort of 168 individuals (see Figure 1 for the study consort diagram). The mean age at the time of injury was 45.3 (SD = 17.8) years, 78% were male, and the average time to the acute care hospital was 3.29 h (SD = 4.07; see Table 2A). On average, participants had 10 neurological exams, with a range of 3–15, over the study period which spanned from 1 to 365 days. Over half (50.6%) had their first exam within 24 h, 84% had their first exam within 48 h, and 94% had their first exam within 72 h. As mentioned previously, in addition to the initial neurological exam in the first week, all participants had at least one exam in the second week post injury. The distribution of neurological severity on admission was 72 (42.9%) AIS A, 33 (19.6%) AIS B, 47 (28%) AIS C, and 16 (9.5%) AIS D (Table 2A). Of the participants who were classified as AIS A on admission, 25.9% converted to AIS B, and 14.8% to AIS C or D by their discharge (Table 2B). More than half (59.4%) of the participants who were classified as AIS B on admission converted to AIS C or D (Table 2B). Further cohort details are included in Tables 2A, 2B.

**Table 2A:** Participant characteristics for the analysis cohort (*n* = 168).

**Variable**	
Age at injury; mean years (SD)	45.3 (17.8)
Male *n* (%)	131 (78)
Mechanism of injury *n* (%)	
Falls	64 (38.1)
Transport	54 (32.1)
Sports	36 (21.4)
Other	14 (8.3)
Charlson Comorbidity Index *n* (%)	
0	136 (81)
1–2	27 (16.1)
3+	5 (2.9)
Injury Severity Score mean (SD)	25.7 (11.6)
Neurological severity of injury on admission (AIS) *n* (%)	
A	72 (42.9)
B	33 (19.6)
C	47 (28)
D	16 (9.5)
Neurological injury level *n* (%)	
High cervical (C1–C4)	80 (47.6)
Low cervical (C5–T1)	88 (52.4)
UEMS change over 14 days post-injury; mean motor score units (SD)	
AIS A	1.7 (4.6)
AIS B	1.07 (5.1)
AIS C	4.3 (7.5)
AIS D	7.1 (11.4)
TMS change over 14 days post-injury; mean motor score units (SD)	
AIS A	1.30 (4.9)
AIS B	3.43 (9.97)
AIS C	9.18 (12.99)
AIS D	16.2 (14.22)
Time to acute hospital; mean hours (SD)	3.29 (4.07)
Surgery *n* (%)	153 (91)
Time of surgery; mean hours (SD)	36.67 (71.62)
Received rehabilitation *n* (%)	147 (87.5)
Acute length of stay; mean days (SD)	56.33 (41.1)
Rehabilitation length of stay; mean days (SD)	144.39 (71.79)

AIS, American Spinal Injury Association (ASIA) Impairment Scale; SD, standard deviation; TMS, total motor score and UEMS, upper-extremity motor score.

**Table 2B:** AIS conversion between admission and discharge.

**Admission AIS**	**Discharge AIS (% conversions)**				
	**A**	**B**	**C**	**D**	**E**
A	48 (59.3)	21 (25.9)	10 (12.3)	2 (2.5)	0
B	2 (5.4)	13 (35.1)	10 (27.0)	12 (32.4)	0
C	0 (0)	3 (5.7)	7 (13.2)	43 (81.1)	0
D	0 (0)	0 (0)	0 (0)	21 (91.3)	2 (8.7)

AIS, American Spinal Injury Association (ASIA) Impairment Scale.

The discharge time for the AIS was mean = 173.34 days, SD = 102.7 days, median = 170.5 days.

**Figure 2:**
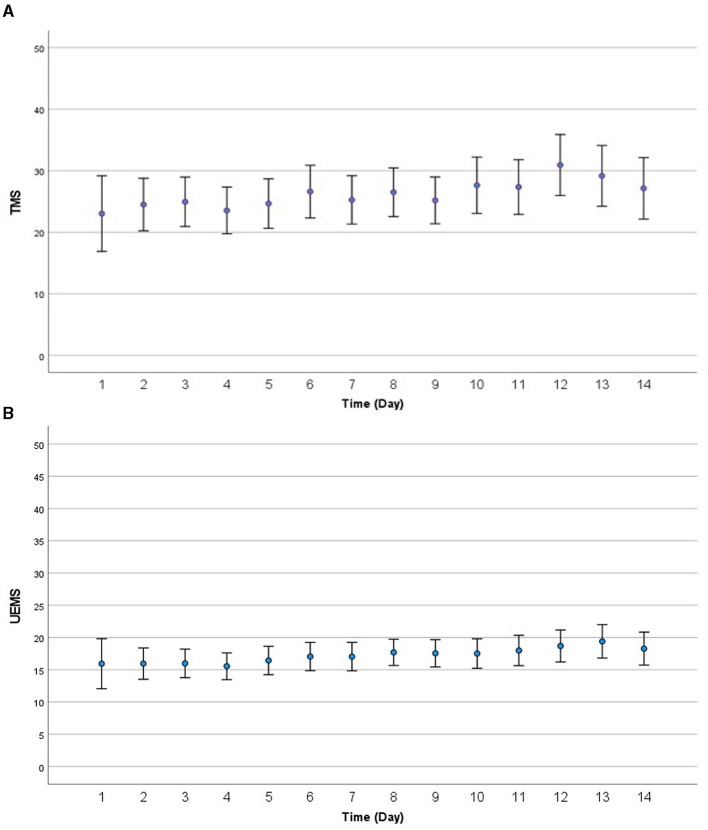
**(A)** Visualization of motor recovery using TMS for the first 14 days after injury for AIS A to D. **(B)** Visualization of motor recovery using UEMS for the first 14 days after injury for AIS A to D. AIS, American Spinal Injury Association (ASIA) Impairment Scale; TMS, total motor score; UEMS, upper-extremity motor score.

**Figure 3:**
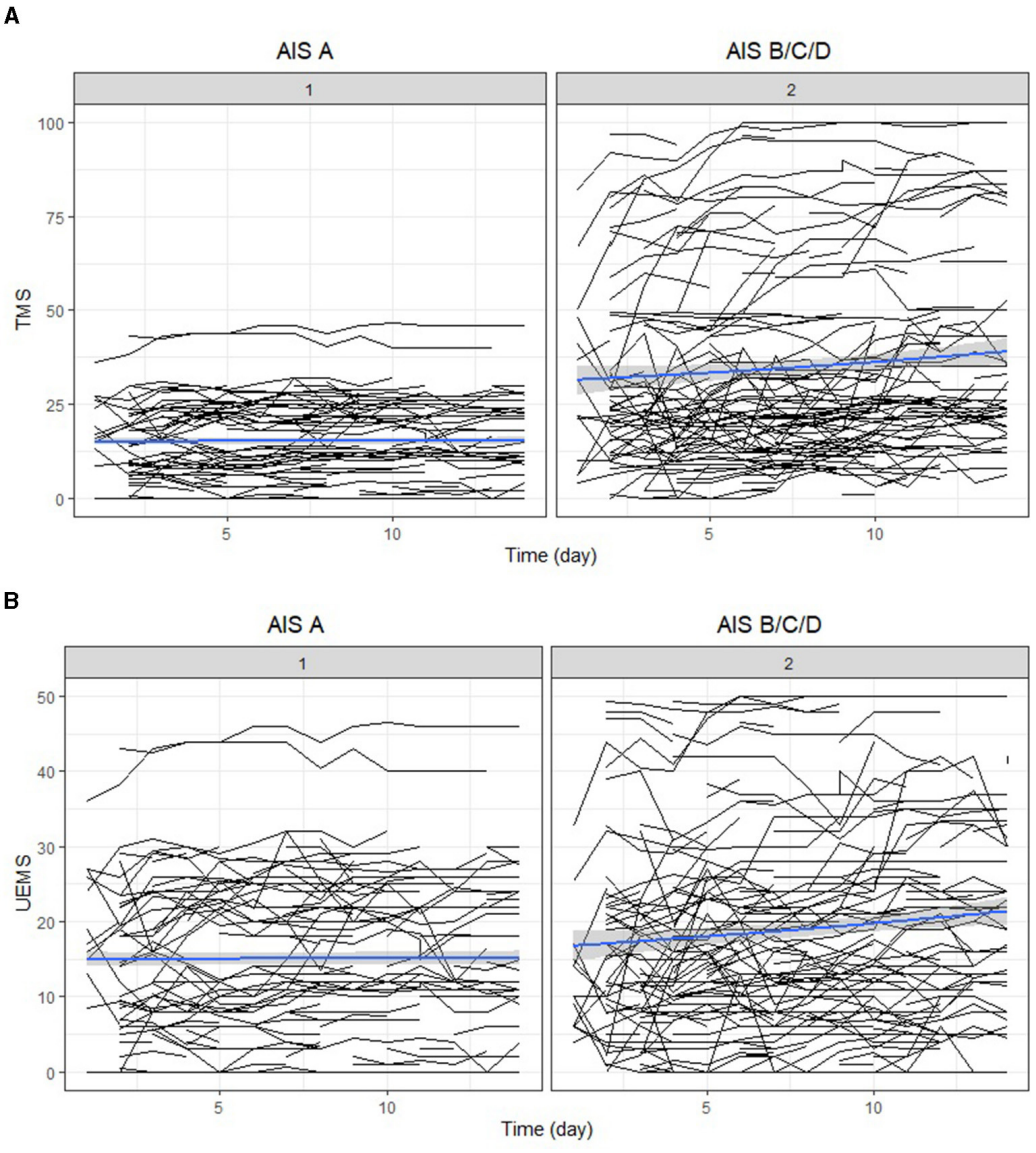
**(A)** Individual trajectory and mean change of total motor score recovery for the first 14 days after injury (Panel 1 AIS A; Panel 2 AIS B, C, D). **(B)** Individual trajectory and mean change of upper-extremity motor score recovery for the first 14 days after injury (Panel 1 AIS A; Panel 2 AIS B, C, D). AIS, American Spinal Injury Association (ASIA) Impairment Scale; TMS, total motor score; UEMS, upper-extremity motor score.

Trajectories of motor recovery starting at 1 day up to 14 days post injury were visualized (see Figures 2, 3). Patterns and amount of motor recovery over the first 14 days were examined using a linear mixed-effect model. For the participants with AIS A injuries, UEMS and TMS were stable and not significantly different over 14 days post-injury. There were significant differences in the change in UEMS and TMS over 14 days for participants with a neurological severity AIS B, C, D, and stratified for neurological level (Table 3A), when compared to individuals with an AIS A injury. The changes in UEMS over 14 days post injury were most pronounced in individuals with AIS D injuries (*p* < 0.001 for UEMS and TMS; Table 3B).

**Table 3A:** Linear mixed effects model for total motor score (TMS) as the outcome.

**Characteristics**	**Entire cohort**	
	**Estimate (95% CI)**	***p*** **-value**
Time, day	0.04 (0.00 to 0.07)	0.04
Time^2^, day	0.00002 (0.00 to 0.00)	0.68
Baseline AIS		
A	Ref	—
B	5.32 (0.06 to 10.58)	0.05
C	15.74 (10.93 to 20.55)	< 0.001
D	64.19 (57.12 to 71.26)	< 0.001
Baseline level of injury		
Upper cervical	Ref	—
Lower cervical	9.60 (5.65 to 13.55)	< 0.001
Baseline AIS ^*^ time, day		
A	Ref	—
B	0.15 (0.07 to 0.22)	< 0.001
C	0.42 (0.35 to 0.48)	< 0.001
D	0.69 (0.52 to 0.87)	< 0.001
Baseline AIS ^*^ time^2^, day		
A	Ref	—
B	−0.0002 (0.00 to 0.00)	0.17
C	−0.0009 (0.00 to 0.00)	< 0.001
D	−0.0067 (−0.01 to 0.00)	< 0.001

**Table 3B:** Linear mixed effects model for upper-extremity motor score (UEMS) as the outcome.

**Characteristics**	**Entire cohort**	
	**Estimate (95% CI)**	***p*** **-value**
Time, day	0.0647 (0.04–0.09)	< 0.001
Time^2^, day	−0.0003 (0.00–0.00)	0.02
Time^3^, day	0.000001 (0.00–0.00)	0.04
Baseline AIS		
A	Ref	—
B	4.50 (0.66–8.34)	0.02
C	3.61 (0.10–7.11)	0.05
D	21.56 (16.43–26.68)	< 0.001
Baseline level of injury		
Upper cervical	Ref	—
Lower cervical	12.22 (9.37–15.07)	< 0.001
Baseline AIS ^*^ time, day		
A	Ref	—
B	0.04 (0.01–0.07)	0.02
C	0.10 (0.07–0.13)	< 0.001
D	0.21 (0.15–0.26)	< 0.001

AIS, American Spinal Injury Association (AIS) Impairment Scale.

Next, we explored the effect of timing of the neurological examination over 14 days, neurological severity (AIS A, B, C, D) and level of injury (high cervical and low cervical) on UEMS and TMS using a mixed-effect model. Specifically, we assessed whether the timing of the first neurological exam had an association with neurological recovery for each of the cervical AIS and neurological level of injury subgroups [i.e., high (C1–C4) vs. low cervical (C5–T1)]. For the cervical AIS A group (high and low cervical), there was no significant effect of timing of the exam on neurological recovery (i.e., motor score change) during the first 14 days post-injury (Table 3A). In the cervical AIS B group, the time of examination significantly impacted the UEMS (4.50; *p*-value = 0.02) and TMS (5.32; *p*-value = 0.05) over 14 days (Figures 2, 3 and Tables 3A, 3B). Furthermore, the effect of time in a linear form as well as a quadratic form (time^2^) were tested in the model and only the linear form (0.15; *p*-value < 0.001) was significant for the AIS B group. For the cervical AIS C group, there were significant differences in UEMS (3.61; *p*-value = 0.05) and TMS (15.74; *p*-value < 0.001) recovery over the first 2 weeks and they were most pronounced around 72 h post-injury. For this group, the timing of the neurological exam was significant in both linear and quadratic form (time, time^2^) using a mixed-effects model. Finally, the results for the AIS D group revealed that the timing of the initial examination was significantly related to changes in both UEMS (21.56; *p*-value < 0.001) and TMS (64.19; *p*-value < 0.001). The AIS D subgroup demonstrated the highest slope of change (i.e., improvement in UEMS and TMS) when compared to the other AIS subgroups. The injury location (high vs. low cervical) and time interaction term was not significant for AIS B, C, and D injuries in the first 14 days post injury.

These results illustrate that the timing of neurological exams and injury severity were important factors. For individuals with the most severe injuries (AIS A group), the timing of the neurological exam did not have a significant impact on the observed neurological recovery. For AIS, B, C, and D injuries, the recovery curve was nonlinear and recovery began immediately after injury, with the most significant changes happening up to 72 h after the injury. Finally, for individuals with the least severe injuries (AIS D group), the timing of the exam was especially important, and they had the highest level of recovery starting at 1 day which continued up to 2 weeks, compared to the other groups. These results demonstrate that individuals who had their first examination at day 1 had more room for improvement than individuals who had their first examination at day 3 post-injury, and the same pattern was observed comparing day 3 to day 14. A general formula based on the regression model is described in the Appendix.

## Discussion

To better understand the neurological trajectory following cervical SCI, we analyzed longitudinal upper-extremity and total motor score data, stratified by neurological severity and level of injury, from day one after SCI up to 14 days. Our results demonstrate that for cervical AIS B, C, and D injuries there is substantial neurological recovery beginning within the first day post-injury and continues up to 14 days post-injury for AIS C and D injuries. Given these changes, clinical studies including subjects with a SCI graded as an AIS B, C, or D should ensure the baseline ISNCSCI assessment for the intervention and control cohorts are completed at the same time post-injury. For cervical AIS A injuries, our results suggest that following the first neurological exam motor score does not change significantly in the first 2 weeks post injury.

Using these models, it is possible to quantify the variation in neurological recovery due to the timing of the examination in the first 14 days following injury which can inform the analysis of registry data or design of clinical trials recruiting participants with a cervical SCI (C1–T1). For example, at one day post injury we can determine the expected natural recovery (e.g., TMS) at 10-day post injury using the regression equation in a patient with a cervical AIS C injury (high and low cervical). The “time” variable is 10 days and after subtracting the TMS from day one (i.e., the motor score at injury) it equals 4.4 and represents the amount of TMS recovery expected at 10 days post injury. A second example includes an individual with an AIS B (high cervical). The “time” variable is 10 days and based on the equation this equals 1.75, which corresponds to the amount of motor score recovery for the AIS B high cervical group at 10 days post injury. Comparing AIS B and C, the effect of time is evident; the change in TMS is 1.75 for AIS B and 4.4 for AIS C. Similar results can also be obtained for UEMS from the linear mixed-effect model.

The literature (see Table 1) reports a large variation in the timing of what is considered a “baseline” examination time and spontaneous neurological recovery is a phenomenon that has likely been underestimated or overlooked previously. Conducting an ISNCSCI exam immediately following injury can be challenging given issues with triage, the need to stabilize the patient and manage polytrauma and a decreased level of consciousness. However, when one considers that “recovery” is measured as the amount of change between an ISNCSCI examination done at a later post-injury time point vs. the ISNCSCI examination done “at baseline,” it is surprising that the timing of that baseline examination and how this might influence the quantification of recovery has not been well studied. In the literature, the recommendation was to conduct a neurological exam any time after 72 h post-injury (28), between 72 h to 1-month post-injury (5, 29) or anywhere between 2 weeks post-injury as the baseline assessment (30). Our findings strongly suggest that individuals who have sustained a SCI (ranging from AIS A to D) should promptly undergo a neurological examination (31), rather than adhering to the commonly recommended practice of scheduling it after the 72-h mark. Even for individuals with AIS A injuries, a number convert to an incomplete injury (AIS B to D) and might have the potential to have significant improvement in motor score (UEMS and TMS). Research studies including individuals (AIS A to D) should have the time of their ISNCSCI neurological exam recorded and be matched to within the same day to ensure the recovery potential is equivalent in studies using SCI registry data as a control or in planning a prospective study (e.g., randomized control trial or observational study) to account for spontaneous recovery. Furthermore, studies should consider using a longitudinal study design rather than a cross-sectional study design. Although a longitudinal study design presents challenges due to the increased cost and time for repeated clinical examinations, it enables a more detailed examination of how the variable(s) of interest change over time, at both the group and individual level. This will allow gradual changes in neurological recovery that may occur in the first few days post injury to be observed.

Results from this study can also be used to “adjust” for differences between the control and intervention group based upon the timing of neurological exams. The regression equations enable the amount of neurological improvement each day post injury to be quantified and so two groups can be matched (e.g., artificially match the intervention group and control group for time of neurological exam). However, further research is needed to validate our results in other countries with SCI registries before these adjustment estimates should be used in future research.

Our previous findings (15) suggested that participants in observational studies should be stratified by neurological severity and level of injury given the heterogeneity of SCI. In this current study where we measured the first 14 days post injury, we were not able to show a difference between upper and lower cervical injuries. However, the effect of level of injury should be explored in studies with larger sample sizes since 43% of our sample has an AIS A injury and this study may be under powered to show a difference between upper and lower cervical injuries. Failure to stratify and/or use an appropriate control group can lead to incorrect conclusions regarding efficacy of a treatment, especially if there are small cohort sizes. Stratification can improve the study efficiency by decreasing the variance and increasing statistical power and so it is suggested individuals are stratified for neurological severity and level of injury. Based on these results, it is also important to record the time of the baseline neurological examination and ensure that control and intervention groups are matched on this variable.

Although this study provides new information on the neurological recovery patterns for cervical SCI, it is important to consider the limitations. Our study examined neurological data reported in days, however, future research should include more precise times reported within hours of injury (e.g., 0–4 h, 4–8 h etc.) and determine if these changes are clinically significant. In this study, AIS conversion was only measured at baseline and at the final neurological exam. As a result, we cannot comment on the timing of the neurological exam as it relates to AIS conversion. Future studies including biomarker and imaging data will provide more precise information on changes in neurological recovery and factors such as age, concurrent injuries, infections, and surgical management that can influence recovery should be considered. In addition, this study focused on cervical injuries and future studies should conduct longitudinal studies in individuals with thoracic and thoracolumbar injuries. Data used in this study is comparable to previous, similar studies examining neurological recovery (8) in participants with cervical SCI, although the number of conversions of AIS A injuries in our cohort is slightly higher (15). This may be due to “spinal shock” which may affect the reliability of neurological examinations at very early timepoints post injury. It is recognized that the concept of “spinal shock” complicates the early assessment of acute SCI patients and can make it quite difficult to discern the true extent of the neurological impairment. This issue is inherently problematic in the clinical evaluation of neuroprotective treatments which must be delivered as soon as possible after injury and therefore do not afford investigators the luxury of just waiting until spinal shock resolves and a reliable neurological examination can be conducted. Our findings highlight the dynamic nature of the injury in the first 14 days, and emphasizes the need to account for the timing of baseline neurological assessment in the interpretation of neurological recovery related to early interventions. Further research into the trajectory of sensory and autonomic scores should also be considered as it is important for neurological and functional recovery as well as further classifying the severity of the spinal fracture using the AO Spine Classification (32).

In summary, we analyzed the trajectories of motor score improvement when multiple examinations were conducted and observed that trajectories are different in the first 2 weeks following a SCI among AIS A, B, C, and D injuries. We demonstrated the need for comparable baseline neurological assessment times within study groups to prevent biasing the interpretation of neurological recovery. These results can help improve the design of future clinical SCI studies by increasing the efficiency, robustness and statistical power (7, 33–35). Future studies should validate these estimates of neurological recovery for the first 14 days in AIS B to D injuries to see if they can serve as an “adjustment factor” to control for any bias due to differences in the timing of the exams.

## Data availability statement

The data analyzed in this study is subject to the following licenses/restrictions: access to deidentified data used for this study is available via the RHSCIR Data Use and Disclosure Policy which is administered by the Praxis Spinal Cord Institute. Requests to access these datasets should be directed to dataservices@praxisinstitute.org.

## Ethics statement

The studies involving humans were approved by Vancouver Coastal Health Research Institute and the University of British Columbia Clinical Research Ethics Board. The studies were conducted in accordance with the local legislation and institutional requirements. Written informed consent was not required from the participants or the participants/next of kin in accordance with local legislation and our institutional requirements.

## Author contributions

NF: Conceptualization, Formal analysis, Writing—original draft. VN: Conceptualization, Writing—original draft, Funding acquisition. ZW: Writing—review & editing. RC-M: Writing—review & editing. CD: Writing—review & editing. CC: Writing—review & editing. TA: Writing—review & editing. ND: Writing—review & editing. SP: Writing—review & editing. JS: Writing—review & editing. CF: Conceptualization, Writing—review & editing. MD: Conceptualization, Writing—review & editing. BK: Conceptualization, Writing—original draft.

## Appendix

We used the statistical models from Tables 3A, 3B to construct the statistical equations for each outcome (TMS and UEMS). The equation below is for TMS, where the Time variable represents the neurological exam time and *Y* represents the respective motor score for that time point.

$$\begin{array}{l} Y_{\text{motor score at any given time}}=\text{Intercept}+{\text{β}}_{1}*\text{Time}+{\text{β}}_{2}*{\text{Time}}^{2}\\ \;+{\text{β}}_{3}*\text{AIS}\;(\text{A}/\text{B}/\text{C}/\text{D})+{\text{β}}_{4}*\text{High cervical}/\text{Low cervical}\\ \;+{\text{β}}_{5}*\text{Time}*\text{AIS}\;(\text{A}/\text{B}/\text{C}/\text{D})+{\text{β}}_{6}*{\text{Time}}^{2}*\text{AIS}\;(\text{A}/\text{B}/\text{C}/\text{D}) \end{array}$$

For a particular timepoint, such as 10 days post-injury, we can forecast the expected natural recovery specifically for that day for each American Spinal Injury Association (ASIA) Impairment Scale (AIS) grades. In this scenario, the Time variable in our equation can be assigned the value of 10 days and the β values used in this equation are derived from the regression model presented in Table 3A for TMS. The β_1_ corresponds to the Time variable, and in our model, it has a value of 0.04. A similar methodology can be applied to predict upper-extremity motor score (UEMS) recovery by utilizing the regression coefficients provided in Table 3B.
